# Supplementary material for: Knowledge, Attitude and Practices towards the Prevention of Schistosomiasis Mansoni in an Endemic Area of Alagoas, Northeast Brazil
Source: Trop Med Infect Dis. 2023 Jan 3;8(1):34. doi: 10.3390/tropicalmed8010034 (PMC9864828; doi:10.3390/tropicalmed8010034)
Supplement: Supplementary file 1 [file tropicalmed-08-00034-s001.zip › tropicalmed-2065594-supplementary.pdf]

Supplementary material

Table S1: Characteristics of knowledge of residents from Feira Grande, Alagoas, Brazil in 2021.

Questions/Alternatives	N	%
**1 - Do you know what schistosomiasis is?**		
No	147	35.68
Yes	265	64.32
**2 - How are people infected with schistosomiasis?**		
Working in the field	84	20.39
Swimming or playing in contaminated water	350	84.95
Walking barefoot	309	75.00
Defecating near water sources	302	73.30
Doing housework in water	309	75.00
Do not know	49	11.89
**3 - Do animals transmit schistosomiasis?**		
No	229	55.58
Yes	183	44.42
**4 - Which animals transmit schistosomiasis?**		
Cat	46	25.14
Dogs	46	25.14
Mosquitoes	23	12.57
Snails	141	77.05
Barber beetles	26	14.21
Do not know	26	14.21
**5 - Can an infected person contribute to the spread of schistosomiasis?**		
No	190	46.12
Yes	222	53.88
**6 - Do you know how to avoid schistosomiasis?**		
No	147	35.68
Yes	265	64.32
**7 - Do you know if there is any specific test for diagnosing schistosomiasis?**		
No	126	30.58
Yes	286	69.42
**8 - How can a person who is or has been infected with schistosomiasis prevent reinfection?**		
Avoiding contact with infected bodies of water and using protective equipment when necessary	348	84.47
Taking antiparasitics every six months	292	70.87
Using the toilet to evacuate stool	311	75.49
Participating in diagnostic and treatment campaigns	329	79.85
Avoiding contact with bodies of water that have any type of snail	337	81.80
Using clean water for drinking and washing	336	81.55
Do not know	34	8.25
**9 - Do you know if there is any specific medicine that treats schistosomiasis?**		

No	105	25.49
Yes	306	74.27
Do not know	1	0.24
**10 - What are the symptoms of schistosomiasis?**		
Fever and headache	156	37.86
Weakness and dizziness	274	66.50
Only water belly	197	47.82
Lack of appetite and weight loss	296	71.84
Enlargement of the liver and spleen	282	68.45
Stomach ulcers	181	43.93
Diarrhea and constipation	290	70.39
Do not know	60	14.56
**11 - Is schistosomiasis endemic in your region?**		
No	48	11.65
Yes	254	61.65
Do not know	110	26.70
**12 - What was your first source of knowledge about schistosomiasis?**		
Public Health Programs	134	32.52
Doctor or Hospital	42	10.19
School	66	16.02
Acquaintances/neighbors	93	22.57
Relatives/older people	92	22.33
None	36	8.74

Table S2: Characteristics of attitudes of residents from Feira Grande, Alagoas, Brazil in 2021.

Questões/Alternativas	N	%
**1 - Do you believe that schistosomiasis can be avoided?**		
No	17	4.13
Yes	395	95.87
**2 - Are you interested in carrying out exams for the diagnosis of schistosomiasis?**		
No	26	6.31
Yes	386	93.69
**3 - Have you ever taken praziquantel through a treatment campaign?**		
No	296	71.84
Yes	116	28.16
**4 - Would you take praziquantel if you were offered it through a treatment campaign?**		
No	12	2.91
Yes	400	97.09
**5 - Do you do anything to protect yourself and your family from schistosomiasis?**		
No	57	13.83
Yes	353	85.68
Did not answer	2	0.49
**6 - How severe do you consider schistosomiasis?**		
Serious	384	93.20
Of some concern	14	3.40

It's not serious	12	2.91
Did not answer	2	0.49

Table S3: Characteristics of practices of residents from Feira Grande, Alagoas, Brazil in 2021.

Questions/Alternatives	N	%
**1 - Do you carry out parasitological examinations of feces every 6 months?**		
Only when doctor requests	349	84.71
No	24	5.83
Yes	39	9.47
**2 - Have you ever performed a specific fecal parasitological examination for the diagnosis of schistosomiasis?**		
No	156	37.86
Yes	225	54.61
Don't know / Don't remember	31	7.52
**3 - Did you relieve yourself (defecate) in open areas in the last 12 months?**		
No	354	85.92
Yes	56	13.59
Don't know / Don't remember	2	0.49
**4 - Have you had regular contact with water from rivers, streams or dams in the region in the last 12 months?**		
No	343	83.25
Yes	68	16.50
Don't know / Don't remember	1	0.24
**5 - Have you used water from rivers, streams or dams in the region for drinking, bathing, washing clothes or washing dishes in the last 12 months?**		
No	359	87.14
Yes	52	12.62
Don't know / Don't remember	1	0.24
**6 - Do you wear protective gear when you have to go into the water at work, for example wellies?**		
No	300	72.82
Sometimes	15	3.64
Yes	91	22.09
Don't know / Don't remember	6	1.46
